# Combined Effects of 2-Methoxyestradiol (Hypoxia-Inducible Factor 1*α* Inhibitor) and Dasatinib (A Second-Generation Tyrosine Kinase Inhibitor) on Chronic Myelocytic Leukemia Cells

**DOI:** 10.1155/2022/6324326

**Published:** 2022-04-28

**Authors:** Yuexian Zhang, Heng Chen, Yunfeng Shen, Xin Zhou

**Affiliations:** ^1^Department of Hematology, Wuxi Branch of Ruijin Hospital, Wuxi, China; ^2^Department of Hematology, Wuxi People's Hospital, Wuxi, China

## Abstract

Chronic myelocytic leukemia (CML) is a frequently encountered type of leukemia in China. Hypoxia-inducible factor 1 (HIF-1) serves as one of the most important factors of oxygen balance transcription. The activation of this gene mostly marks a poor outlook for cancer patients. To clarify the therapeutic effect of inhibiting this gene on CML, the present study is aimed at exploring the treatment effects of 2-methoxyestradiol (2-ME2), dasatinib alone, and combined both on K-562 cells and the possible mechanism of 2-ME2 in treating the disorder. The levels of HIF-1*α*, vascular endothelial growth factor (VEGF), and glutamate synthase 1 (GLU1) genes in K-562 cells were affected dose-dependently after 2-ME2 administration. 2-ME2 induced cell apoptosis by downregulating antiapoptotic protein expressions of Bcl-xl and Bcl-2. The therapeutic effect of single 2-ME2 was superior to single dasatinib, and the effect of combined therapy of both drugs produced better effectiveness than either of the single drug. Once the concentration of 2-ME2 exceeded 0.5 *μ*M, downregulated C-myc gene expression could exert roles in anti-CML cell proliferation and inducing apoptosis. Dasatinib might participate in the inhibition of the C-myc pathway during this process whereas its effect remained not clear. Taken together, abnormal high expression of HIF-1*α* exerted an essential role in CML occurrence and development. Inhibition of this gene could markedly increase cell apoptosis in a dose-dependent fashion. Moreover, 2-ME2 could induce cell apoptosis by downregulating the C-myc gene and exert an apoptotic effect by downregulating Bcl-xl and Bcl-2 which act as antiapoptotic proteins.

## 1. Introduction

CML represents a clonal malignant disease due to the hematopoietic stem cell proliferation of bone marrow [1]. This tumor is characterized by abnormal elevation of peripheral white blood cells. Tyrosine kinase inhibitor (TKI) is introduced as a targeted therapy drug, namely, imatinib, nilotinib, and dasatinib, which benefits 85% to 90% of CML patients with a 10-year survival rate. Unfortunately, the extensive administration of TKI has brought about increasing drug resistance problems in CML patients [2]. Therefore, it is of vital significance to explore novel therapeutic targets and targeted medication for CML to further improve the therapeutic level of CML.

As a kind of oxygen balance transcription factor, HIF-1 presents ubiquitously in human cells and can be activated in various hypoxic environments, thereby ensuring the body to better adapt to the external hypoxic environment. HIF-1 is aggregated by two subunits HIF-1*α* and HIF-1*β*. The expression of the active HIF-1*α* increases when the body is in a hypoxia condition, while HIF-1*β* is a constitutive subunit, and it can be expressed in both normoxic and hypoxic environments. HIF-1*α* accumulates in the nucleus after transcriptional activation, combines with HIF-1*β* transferred from the cytoplasm to the nucleus, and forms a dimer which is an active HIF-1 complex. It combines with the hypoxia response element (HRE) of downstream target genes VEGF and erythropoietin (EPO) to be regulated and forms a transcription initiation complex to initiate the transcription of target genes. As the activation of HIF-1*α* in a relatively hypoxic environment, it further activates multiple downstream target genes, promotes the proliferation of tumor cells, and ensures energy metabolism of tumor cells, thereby promoting tumor angiogenesis and ultimately accelerating the tumor's growth, metastasis, and drug resistance. There are abundant researches on the impact of HIF-1*α* on solid tumors at home and abroad. Numerous studies believe that HIF-1*α* acts as a risk factor for determining tumor prognosis and a vital antitumor target. HIF-1*α* is overexpressed in several cancer tissues including liver cancer, lung cancer, osteosarcoma, glioma, ovarian cancer, and renal cell carcinoma and regulates biological behaviors covering angiogenesis, tumor cell metabolism, and immune escape of tumor stem cells. In the leukemia microenvironment, all abnormalities indicate the presence of HIF-1 response, including the elevation of secretion of VEGF, C-X-C motif chemokine ligand 12 (CXCL12) and stem cell factor (SCF), hypoxia, and acidity. Rouault-Pierre et al. and Radwan et al. have confirmed that the bone marrow of acute lymphocytic leukemia (ALL) patients is highly hypoxic in the advanced leukemia stage by experiments, and all patients presented overexpressed HIF-1*α* at the time of definite diagnosis [3, 4]. When there is a cytogenetic response, the positive rate of HIF-1*α* staining is markedly reduced. Wang et al. have explored the role of HIF-1*α* and VEGF-A genes in acute myeloid leukemia (AML) by collecting samples of peripheral blood and bone marrow of 57 AML patients and 17 healthy subjects [5]. q-PCR has been performed to detect the expressions of HIF-1*α* and VEGF-A, and the results indicate that the expressions of HIF-1*α* and VEGF-A in AML patients are higher than those of normal control, and HIF-1*α* has a positive correlation with VEGF-A. They believe that this may be related to the growth of leukemia cells and may become a promising therapeutic target. Kontos et al. have found that chronic lymphocytic leukemia (CLL) patients having high HIF-1*α* mRNA expressions have a lower overall survival rate, and the significance of the expression levels applied for prognosis guidance can be independent of clinical stage and other prognostic indicators [6]. Hence, overexpression of HIF-1*α* may serve as an independent molecular marker of poor prognosis in CLL. Abdul-Aziz1 et al. have noted that hypoxia can cause high expression of migration inhibitory factor (MIF) in AML cells in bone marrow, and the expression level is higher than that of peripheral blood or spleen [7]. Their research on AML has proved that silenced HIF-1*α* under hypoxic conditions can inhibit the transcriptional regulation of MIF, thereby improving the survival rate of AML models, indicating that the hypoxia/HIF-1*α*/MIF axis is of vital significance in promoting survival and proliferation of AML tumors. Zhe et al. have also demonstrated the overexpression of HIF-1*α* in AML. Its inhibitor 2-ME2 inhibits HIF-1*α* and markedly induces apoptosis in AML cells [8]. The inhibitor works superior to traditional chemotherapeutics, namely, cytarabine. Meanwhile, 2-ME2 presents antileukemia activity in hematogonium of AML patients instead of normal cells. As a consequence, 2-ME2 may represent a potential therapy for AML patients. And HIF-1*α* inhibitor may be introduced as a novel therapeutic drug. By conceiving the present project, we expect HIF-1*α* inhibitor to play a role in CML.

This study selected 2-methoxyestradiol (2-ME2) as an experimental agent. As a natural estrogen metabolite, it mainly targets HIF and is under a clinical trial at phase I/II. Despite the fact that it inhibits HIF-1*α*, microtubules, and tumor angiogenesis and has a role in antiproliferation and antiangiogenesis, the role and mechanism of 2-ME2 in CML are still unclear. This study initiated to clarify the expression of HIF-1*α* and downstream target genes in CML cells, elucidate the combined effect of the HIF-1*α* inhibitor 2-ME2 and the second-generation TKI dasatinib on CML cells, and further demonstrate the possible action mechanism of the combined therapy of both agents. Through this research, we can further recognize the role of HIF-1*α* including downstream genes in CML and explore new targets and targeted drugs for CML treatment. It can also give hope to CML patients who have no drug available by designing HIF-1*α* inhibitor 2-ME2 in combination with existing TKI drugs.

## 2. Materials and Methods

### 2.1. Cell Culture and Drug Solution Preparation

Human chronic myeloid leukemia cells (K-562 cells) were provided by the Cell Bank of the Chinese Academy of Sciences and subsequently cultured in 15% fetal bovine serum + 1%double antibodies + IMDM medium.

*2-ME2 solution preparation*: according to the instructions (MedChemExpress, USA), 10 mg of 2-ME2 drug powder was weighed and dissolved in 3.3068 mL DMSO to prepare a 10 mM 2-ME2 mother liquor, which is available at -20°C in 1 month. The liquor can be diluted with IMDM medium for later use.

*Dasatinib solution preparation*: as per the instructions (MedChemExpress, USA), 1 mg of dasatinib powder was weighed and dissolved in 2.0491 mL DMSO to prepare a 1 mM dasatinib mother liquor, which is available at -20°C in 1 month. The liquor can be diluted with IMDM medium for later use.

### 2.2. CCK-8 Detection of the Survival and Inhibition Rates of Cells

Cells in the logarithmic growth stage were taken by which cell suspension at a concentration of 4 × 10^5^/mL was prepared and inoculated in 96-well culture plates. An appropriate amount of the drug solution was added to the 96-well plates to make a 100 *μ*L total volume containing cell suspension and drug solution at a certain drug concentration. The negative control group was supplemented with cell suspension only, and the blank control was added with a complete medium only. The 96-well plates were set at different drug concentrations, each group set multiple wells, and the outermost circle of the 96-well plates was added with PBS. After 24 h incubation, each well was added with 10 *μ*L CCK-8 cell proliferation/toxicity detection reagent and continued incubation for an additional 3 h. The M5 multifunctional microplate reader was employed to detect absorbance value at 450 nm. After removing the highest and lowest absorbance values, an average value was obtained for the multiple-well data. Cell survival rate% = (absorbance of experimental wells − absorbance of blank wells)/(absorbance of negative control wells − absorbance of blank wells) × 100%; cell inhibition rate% = (absorbance of control well − absorbance of experimental well)/(absorbance of control well − absorbance of blank well) × 100%. GraphPad software (Version 9.0, USA) was applied to calculate the curve of proliferation inhibition, 24 h cell inhibition rate, cell survival rate, and IC50 value.

### 2.3. Western Blot Detection of the Target Gene Expression in K-562 Cells at the Protein Level

*Cell preparation*: K-562 cells were harvested at the logarithmic growth phase reaching 1 × 10^6^ cells and inoculated into six-well plates. Different concentrations of drug solutions were supplemented to make the total volume of each well 3 mL. The control group was given no drugs and cultured at 37°C, 5% carbon dioxide condition for 24 h. After 24 h, centrifugation was performed at 1,000 rpm for 5 min before removal of the supernatant. Cell pellets at the bottom were collected and cleaned twice using PBS solution for subsequent centrifugation again with the same condition and time period to obtain cell precipitate.

*Extraction of total cell protein*: following centrifugation at 1,000 rpm for 5 min, the treated K-562 cells were harvested after two cycles of washing using PBS to obtain the pellets; RIPA lysis solution, phosphatase, and protease inhibitors were blended at a 99 : 1 : 1 rate based on the instructions and let stand on ice for 5 min. The mixed solution was supplemented to the collected cell precipitate, gently blown into bubbles with a pipette tip (70 *μ*L of the mixed solution was supplemented to every 10^6^ cells), and let stand on ice for 30 min. Following centrifugation at 12,000 rpm at 4°C for 15 min, the supernatant was obtained. The protein concentration was subjected to the BCA method. Then, an appropriate amount of protein tracer loading buffer (5x) was added to the protein samples at a 4 : 1 rate, vortexed evenly, and boiled at 100°C for 10 min.

*Western blotting*: firstly, 8% separation gel and 5% concentrated gel were prepared. Based on actual protein concentrations, the actual sample volume was calculated at a sample volume of 50 *μ*g per well, and the marker and the sample were added to the wells in turn. The voltage was set at 80 V for gel running. When the sample band was at the lower separation gel, the voltage was switched to 120 V. The PVDF membrane was trimmed to the same size as the gel and placed in methanol for activation. The filter mesh and filter paper were immersed in the transfer buffer solution, the transfer film clipboard was turned downward, the current was adjusted to 300 mA, and the membrane was transferred in ice water at a constant current for 2 h. After transfer, the membrane was placed in a small chamber containing 5% skimmed milk powder and kept on a shaker for 1 h at room temperature. Primary antibody solution was prepared in proportions with primary antibody diluent as per the instructions, and secondary antibody solution was prepared in proportions with 1x TBST based on the antibody instructions. After blocking, the membrane was washed 3 times on a shaker using 1x TBST, 10 min each time. Subsequently, based on the size of the protein marked by the marker, the membrane was trimmed at the required molecular weight, soaked in the prepared primary antibody solution, and incubated overnight on a 4°C shaker. After the primary antibody incubation was completed, the membrane was rinsed 3 times with 1x TBST, 10 min each time. After membrane washing, the prepared secondary antibody solution was supplemented to the chamber and cultured for at least 1 h. TBST was used for washing 3 times, 10 min each time. A luminescent liquid was prepared at a 1 : 1 ratio and mixed well. The membrane was horizontally placed on the luminescent board with the surface upward. The luminescent liquid was evenly dripped for exposure. The obtained bands were obtained using the ImageJ software (Version 1.8.0, USA).

### 2.4. Detection of the Expression of the Target Gene in K-562 Cells by Real-Time Fluorescent Quantitative PCR Technology at the Molecular Level

*RNA extraction*: after treating the cells with drugs, the cell precipitate was transferred to a 1.5 mL enzyme-free EP tube and centrifuged at 2,000 rpm at 4°C for 5 min to remove the supernatant. 1 mL TRIzol was added, blown evenly, lysed on ice for 5 min, and centrifuged as previously described. The supernatant was collected after centrifugation and delivered to another EP tube. The tube was supplied with 200 *μ*L chloroform, and the solution was mixed violently on a vortexer, let stand for 5 min, and centrifuged under the same conditions again. After centrifugation, the upper layer of water was collected and delivered to a new EP tube, added with an equal volume of isopropanol, mixed well, let stand at room temperature for 10 min, and centrifuged at 12,000 rpm 4°C for 10 min. Following the removal of the supernatant, the pellets were collected. 1 mL 75% ethanol was added, placed upside down, and centrifuged at 7,500 rpm 4°C for 5 min. After the supernatant was removed, the precipitate was dried in a ventilated place for 20 min. Then, an appropriate amount of DEPC water was supplied to dissolve the precipitate. A spectrophotometer was applied to determine the concentration and OD value of the extracted RNA samples (A260/280).

*cDNA reverse transcription*: gDNA was removed to prepare a 20 *μ*L reaction system on ice as the following: 2 *μ*L 5x gDNA Eraser Buffer, 1 *μ*L gDNA Eraser, total RNA, and 1 *μ*g RNA. RNase Free dH_2_O was supplied, and the total volume of the reaction solution was 10 *μ*L. 1 *μ*g RNA was supplemented to each system; the volume of RNA was calculated based on the measured RNA concentration. After adding the reagents, the solution was let stand at room temperature for 5 min.

*Reverse transcription reaction*: the reaction system of 20 *μ*L was made on ice (removal of the reaction solution from 10 *μ*L gDNA, 1 *μ*L PrimeScript RT Enzyme Mix I, 1 *μ*L RNase Free dH2O, 4 *μ*L RT Primer Mix, and 4 *μ*L 5x PrimeScript Buffer 2 at 20 *μ*L in total). After being mixed evenly, the solution was loaded to the machine which was configured as per the reagent manual.

*Fluorescence quantitative PCR reaction*: 20 *μ*L of the real-time fluorescent quantitative PCR reaction system was processed on ice using 10 *μ*L TB Green Premix Ex Taq II, 0.8 *μ*L PCR Forward Primer, 0.8 *μ*L PCR Reverse Primer, 0.4 *μ*L ROX Reference Dye II, 2 *μ*L DNA template, and 6 *μ*L sterilized water at 20 *μ*L in total. Each of the samples had four replicate wells, and the operation method referred to the Applied Biosystems 7500 Fast Real-Time PCR System. A two-step method was employed for real-time fluorescence quantitative PCR reaction.

### 2.5. Flow Cytometry Detection of K-562 Cell Apoptosis after 24 h Drug Treatment

*Cell treatment*: the cells were collected by centrifugation at 1,000 rpm for 5 min and counted. The cell concentration was set to 5 × 10^5^/mL. Subsequently, 2 mL of cell suspension was added in six-well plates and added with different concentrations of drug solution and culture medium, so that the total volume of each well was 3 mL, and cultured at 37°C with 5% CO_2_ for 24 h.

The cells were double-stained using annexin V and propidium iodide (PI), and cell apoptosis was detected using flow cytometry. After drug administration at different concentrations for 24 h, the cells were collected for centrifugation at 1,000 rpm for 5 min, and the supernatant was abandoned. The cells were resuspended using PBS, and the cell concentration was adjusted at 1 × 10^6^/mL. Of 0.5 mL, the previously described cell suspension was taken and centrifuged at 1,000 rpm for 5 min, and the supernatant was abandoned; cell resuspension was added with 0.5 mL Binding Buffer and 10 *μ*L FITC-labeled annexin V, mixed well, and incubated for 30 min at room temperature away from the light. Then, 5 *μ*L PI was added, mixed well, and incubated at 4°C for 3 min in the dark. After incubation, 500 *μ*L Binding Buffer was provided and immediately analyzed on a flow cytometer.

### 2.6. Detection of Effects of 2-ME2 Combined with Dasatinib on CML

To elucidate the inhibitory effects of a single drug and combined drugs on K-562 cells, K-562 cells were classified into the following four groups. The first one was blank control, to which only complete medium was added; the second group was the 2-ME2 single drug group, to which the 2-ME2 solution was added to the K-562 cell suspension, so that the final concentration of the drug was IC50 at 9.4 *μ*M; the third group was the dasatinib single drug group, which dasatinib solution was added to K-562 cell suspension to make the final concentration of the drug IC50 (according to the drug instructions, the IC50 value of dasatinib was 1.30 nM); the fourth group was the combination of 2-ME2 and dasatinib. The K-562 cell suspension was added with 2-ME2 solution and dasatinib solution to make the 2-ME2 drug at the final concentration of 1/2 IC50 (4.7 *μ*M) and dasatinib at 1/2 IC50 (0.65 nM). After being incubated for 24 h, the cells were harvested for experimentation.

Furthermore, apoptosis of K-562 cells was detected after combined administration of 2-ME2 and dasatinib by flow cytometry. K-562 cells were classified into three groups: The first was the single dasatinib group at concentrations of 0, 0.45, 0.6, 0.9, and 1.2 nM, respectively. The second group was the single 2-ME2 group at concentrations of 0, 3.25, 4.7, 7.05, and 9.4 *μ*M, respectively. The third group was the two-drug combination group at concentrations of 0, 1/4 IC50, 3/8 IC50, 1/2 IC50, and 3/4 IC50. After culture of 24 h, the cells were obtained for experimentation.

### 2.7. Statistical Analysis

SPSS 17.0 software was adopted for data analysis and GraphPad Prism 9.0 for statistical analysis and graphing. The results were expressed as the mean ± standard error (*X* ± SEM). Mean analysis of multiple groups was subjected to one-way or two-way ANOVA methods. The values of *P* < 0.05 indicated that the difference was statistically significant.

## 3. Results

### 3.1. The Expression of HIF-1*α* and Downstream Target Gene in CML

**Figure 1:**
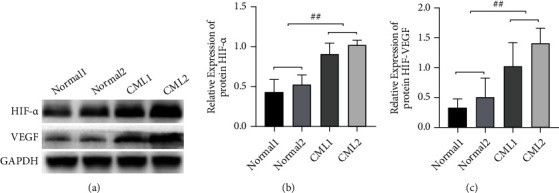
Expressions of HIF-1*α* and VEGF proteins in the bone marrow of healthy individuals and CML patients: (a) Western blot assays of protein expression; (b) HIF-1*α* protein gray values; (c) VEGF protein gray values. ^##^*P* < 0.01.

Firstly, we detected the levels of HIF-1*α* and VEGF in CML bone marrow patients. Comparison of the relative protein levels of HIF-1*α* and VEGF, a downstream target gene, was conducted between two cases of relatively normal and two cases of CML patients using Western blot. The protein levels of HIF-1*α* and VEGF in bone marrow samples of CML patients were markedly higher than those of relatively normal patients versus the normal control group (*P* < 0.05, Figure 1), and the difference was statistically significant. The previously stated results indicated that the abnormally high expression of HIF-1*α* and VEGF might regulate the bone marrow microenvironment of CML patients.

**Figure 2:**
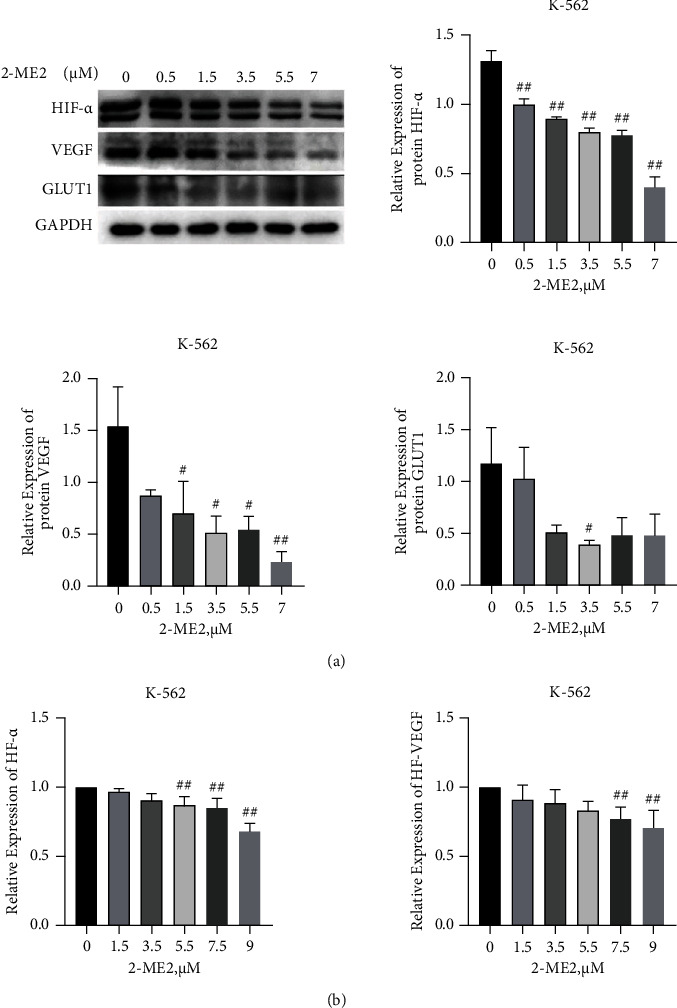
After 2-ME2 treatment of K-562 cells for 24 h, the expression of downstream target genes was detected. (a) Western blot detection of HIF-1*α*, VEGF, and GLUT1 protein expressions and gray values. (b) qPCR detection of the mRNA expressions of HIF-1*α* and VEGF. Compared with control group, ^#^*P* < 0.05; ^##^*P* < 0.01.

As marked overexpression of HIF-1*α* was visualized in CML bone marrow patients, the HIF-1*α* inhibitor 2-ME2 was then employed to treat K562 cells thereby evaluating responses of HIF-1*α* and the downstream target gene. K-562 cells were administered 2-ME2 at different concentrations (the final concentration of the drug solution was at 0.5, 1.5, 3.5, 5.5, and 7 *μ*M) for 24 h, and a control group was set (no drug administration) for the experiment. Western blot detection revealed that as the concentration of HIF-1*α* inhibitor 2-ME2 increased, the protein expressions of HIF-1*α* and downstream target genes VEGF and GLUT1 decreased. Both protein expressions of HIF-1*α* and VEGF presented in a dose-dependent manner show obvious antiproliferative activity (*P* < 0.05, Figure 2(a)), and the statistical difference was significant. RT-qPCR showed that the expression levels of HIF-1*α* and VEGF mRNA also showed a downward trend after treatment with different concentrations of 2-ME2 (*P* < 0.05, Figure 2(b)).

### 3.2. The Effect of HIF-1*α* Inhibitor 2-ME2 on the Proliferation and Apoptosis Pathway of CML Cells

**Figure 3:**
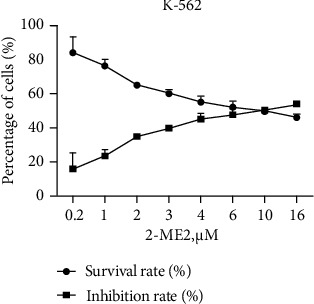
Cell survival rate and inhibition rate of K-562 cells treated with 2-ME2 for 24 h.

To determine the 24 h IC50 value of 2-ME2 in K-562 cells, a dose-response experiment was performed. K-562 cells were incubated for 24 h with increasing 2-ME2 doses (the final concentrations of 2-ME2 at 0.2, 1.0, 2.0, 3.0, 4.0, 5.0, 6.0, 10.0, and 16.0 *μ*M, respectively). The CCK-8 method was employed to detect the survival rate and inhibition rate of cells, and the effect on cell survival and inhibition was evaluated by curve plotting. The test results of CCK-8 showed that as the concentration of 2-ME2 increased, the inhibition rate of K-562 cells gradually increased whereas the survival rate gradually decreased (*P* < 0.05). The IC50 value of K-562 cells following 24 h 2-ME2 treatment was calculated as 9.4 *μ*mol/L using the fitted curve (Figure 3).

**Figure 4:**
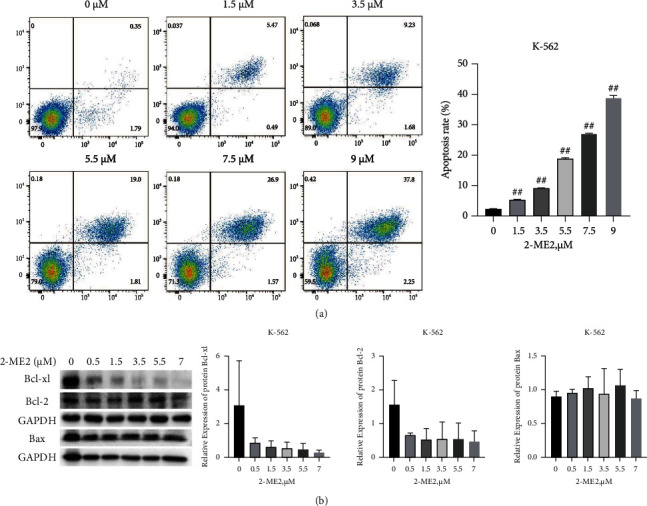
Apoptosis of K-562 cells after treatment with 2-ME2 for 24 h. (a) Flow cytometry detection of cell apoptosis. (b) Western blot detection of Bcl-xl, Bcl-2, and Bax protein expressions. Compared with control group, ^##^*P* < 0.01.

To further clarify the effect of 2-ME2 on K-562 cell apoptosis, the cells were cultivated at different concentrations of 2-ME2 (the final concentrations of 2-ME2 were 0, 1.5, 3.5, 5.5, 7.5, and 9 *μ*M, respectively) 24 h. After double-staining was performed using annexin V and PI, cell apoptosis was detected via flow cytometry. The results of flow cytometry indicated that the higher the concentration of 2-ME2, the higher the apoptosis rate under the condition of basically the same cultivation environment (*P* < 0.05, Figure 4(a)). Moreover, after the concentration was greater than 3.5 *μ*mol/L, the increase in the apoptosis rate increased markedly.

To clarify the related mechanism of 2-ME2 in inducing apoptosis and inhibiting proliferation of K-562 cells, the cells were cultivated with different concentrations of 2-ME2 (the final concentrations of 2-ME2 were 0, 0.5, 1.5, 3.5, 5.5, and 7 *μ*M, respectively) and collected 24 h for subsequent experimentation. Western blot detection results showed that 2-ME2 downregulated the protein expressions of antiapoptotic proteins Bcl-2 and Bcl-xl (*P* < 0.05), while the protein expression of proapoptotic protein Bax did not change significantly (*P* > 0.05, Figure 4(b)).

### 3.3. Action of 2-ME2 Combined with Dasatinib on CML

**Figure 5:**
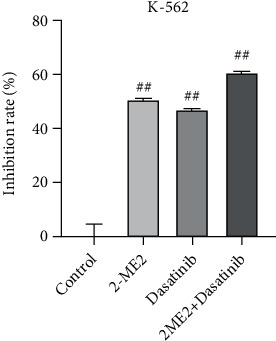
The inhibitory rate detection of K-562 cells 24 h after single drug administrations and combined administration. Compared with control group, ^##^*P* < 0.01.

CCK-8 findings showed that the cell inhibition rate of the single 2-ME2 group was 50.7%, that of the single dasatinib group was 23.2%, and that of the two-drug combination group was 60.7%. The effect of the single 2-ME2 drug group was superior to the single dasatinib group, and the two-drug combination group was better than both single drug groups (*P* < 0.05, Figure 5).

**Figure 6:**
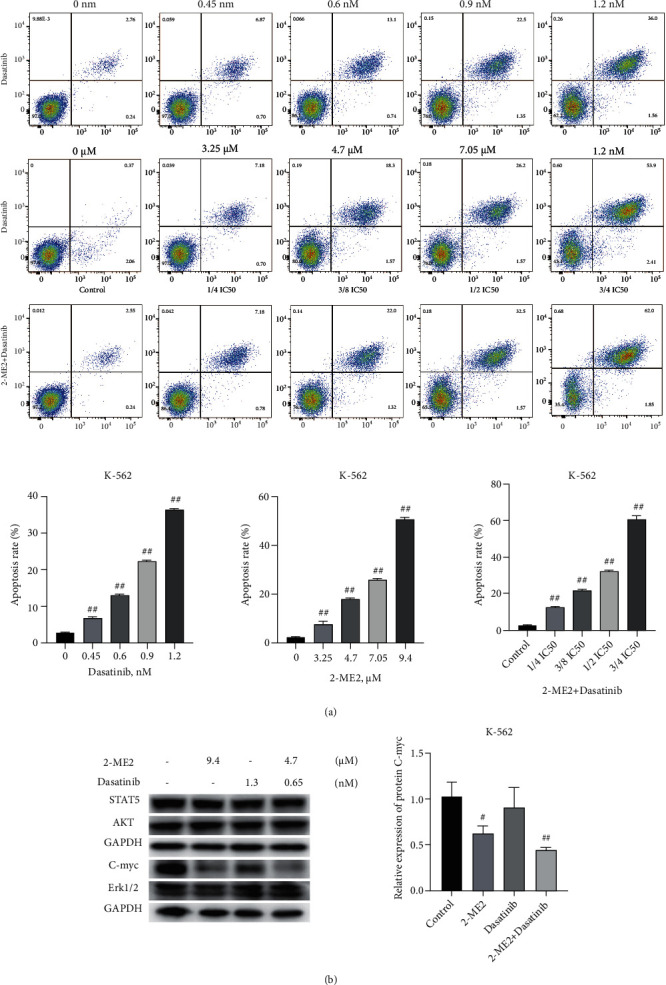
The effects on K-562 cell apoptosis and the regulation of related pathways after 24 h of single and combined administration: (a) flow cytometry detection of cell apoptosis; (b) Western blot detection of protein expressions. Compared with control group, ^#^*P* < 0.05; ^##^*P* < 0.01.

The results of flow cytometry showed that apoptosis of K-562 cells after administration of single dasatinib and single 2-ME2 were dose-dependent, and the higher the drug concentration, the greater the apoptosis. However, there was no linear relationship between concentration and apoptosis. In general, 2-ME2 monotherapy induced a higher rate of apoptosis than dasatinib. Secondly, after being administered the combined drugs, the induction rate of apoptosis of K-562 cells was apparently increased, compared with the single drug therapy at approximately the same concentration. Besides, the effect of combined therapy was more satisfactory than either of the single administration, which was consistent with the CCK-8 assay result (*P* < 0.05, Figure 6(a)).

The previously described experimental results revealed that combined administration therapy achieved a better effect than both single drug treatments. To clarify the inhibitory mechanism of the single drug and the combination therapy on K-562 cells, the experimental cells of the first part in the results section were collected again for verification. Western blot detection results indicated that 2-ME2 could inhibit the expression of C-myc protein, causing its downregulation (*P* < 0.05), without affecting the expression of ST*Α*T5, AKT, and Erk1/2 pathways. Among them, dasatinib might be involved in the inhibition of the C-myc pathway, but its role was not clear (Figure 6(b)).

**Figure 7:**
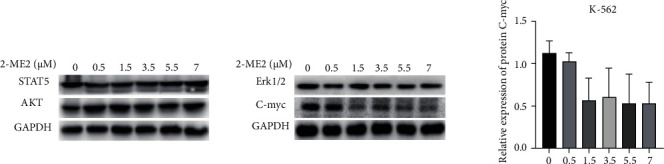
The expression of related pathways in K-562 cells treated with 2-ME2 for 24 h detected by Western blot.

The above experiments revealed that the expression of C-myc protein could be inhibited and downregulated with single 2-ME2 application, but dasatinib alone produced no effect on it. To verify the mechanism of 2-ME2 inducing apoptosis of K-562 cells, the relevant experiments were performed for further assessment. K-562 cells were cultivated at different concentrations of 2-ME2 (the final concentrations of 2-ME2 were 0, 0.5, 1.5, 3.5, 5.5, and 7 *μ*M, respectively) and collected after 24 h for subsequent experimentation. The results of Western blot showed that in the process of 2-ME2 acting on K-562 cells, downstream pathways such as STAT5, AKT, and Erk1/2 did not affect much during the process, while C-myc was significantly downregulated. And 0.5 *μ*M concentration of 2-ME2 had no obvious effect on the C-myc gene. When the concentration continued to increase, the expression of C-myc gene was significantly reduced, but there was no significant dose dependence (*P* < 0.05). It is speculated that the effect of 2-ME2 on C-myc gene was in a phased manner. When the concentration reached the next platform, the expression of C-myc gene will change further. This process verified the downregulation of C-myc gene in K-562 cells by 2-ME2 (Figure 7).

## 4. Discussion

CML is a disease caused by abnormal pluripotent hematopoietic stem cells. It is featured by the existence of t(9;22)(q34;q11) translocation of the Philadelphia Chromosome (Ph chromosome). The translocation contributes to the proliferation of leukemia cells, and when the BCR-ABL tyrosine kinase was activated on the Ph chromosome, it initiates disease development in three phases [9]. The establishment of the first generation of TKI imatinib as the standard therapy for CML marks the beginning of a new era using CML treatment [10, 11]. It is a small molecule protein tyrosine kinase inhibitor that competitively binds to the ATP binding site on the BCR-ABL protein, thereby blocking the continuous phosphorylation of ABL kinase and its downstream molecules and inducing apoptosis in CML cells. Compared with imatinib, the second-generation TKI dasatinib can induce hematological and cytogenetic responses in CML or Ph-positive ALL patients, which realizes an earlier and better remission of the disease conditions, and the rate of progression to the accelerated or blastic phase is minimized. Moreover, the drug is well tolerated and may be eligible to discontinue the use of tyrosine kinase inhibitors [12, 13].

There are mainly two subunits of HIF-1: HIF-1*α* and HIF-1*β*. The former one acts as the only oxygen regulating subunit, which can regulate the metabolism from oxidative stress to glycolysis by stimulating expressions of glycolytic enzymes, glucose transporters, and glycolysis inducing factors [14]. HIF-1*α* can also regulate hypoxia response both in normal tissues and cancer cells. Combined with HIF-1*β* under hypoxia, it regulates the transcription and expression of several hypoxia-responsive genes. The oxygen partial pressure (PO_2_) in the air is 160 mmHg, which is significantly higher than that in the pulmonary veins of 80-100 mmHg. The PO_2_ in different organs is the result of the balance of oxygen delivery and consumption to different tissues. When the blood reaches the bone marrow, the PO_2_ is about 49 mmHg; an oxygen gradient is developed throughout the skeletal system, which is vital and beneficial to cell physiology [3]. Studies have shown that the presence of leukemia stem cells (LSC) under an extremely hypoxic microenvironment is called “bone niche,” and HIF-1*α* expression might be increased. Conversely, the vascular niche is located near the endothelial tissue, at a relatively low degree of hypoxia, and the expression level of HIF-1*α* is also low. These different areas are believed to be related to the different stages of hematopoiesis. Hypoxic inner bone niches support hematopoietic stem cells at quiescence and immaturity, while vascular niches stimulate cell proliferation and maturation. To clarify the hypoxia state and HIF-1*α* expressions of CML patients, we firstly collected bone marrow specimens from two CML patients and two healthier patients. Detection results of Western blot revealed that levels of HIF-1*α* and VEGF mRNA in the bone marrow microenvironment of CML patients were elevated compared with those of relatively normal patients, indicating that HIF-1*α* and the downstream target gene VEGF were of vital significance in the hypoxic bone marrow microenvironment. Secondly, we described the molecular activity of 2-ME2 in the CML cell line (K-562 cells). VEGF and GLU1 are two target genes of HIF-1*α*; they intimately link to the growth and progression of solid and hematological tumors. HIF-1*α* inhibitor 2-ME2 was introduced to treat K-562 cells to construct a low/lost expression of HIF-1*α*. Results of Western blot revealed that protein expression levels of HIF-1*α* and downstream target genes VEGF and GLU1 were markedly reduced in K-562 cells. Meanwhile, HIF-1*α* and VEGF were dose-dependent. The higher the concentration of 2-ME2, the lower the expression of the gene. This effect increased the antiproliferative activity of 2-ME2 on K-562 cells to a certain extent. At the transcription level, HIF-1*α* and VEGF target genes also showed a downward trend, which was in agreement with the results of Western blot detection. On the one hand, the above results indicated that HIF-1*α* plays a role by regulating the expression of downstream target genes. On the other hand, it also suggested that the bone marrow microenvironment of CML patients is in an actually extreme hypoxic state. Several reasons were hypothesized for this situation as the following: (1) The bone marrow cavity is a closed space. The oxygen partial pressure within the cavity is unevenly distributed. When CML leukemia cells proliferate in large numbers, they occupy most part of the bone marrow cavity and increase energy consumption, which will inevitably lead to cell hypoxia. (2) Proliferation of a large number of leukemia cells limits the growth of normal red blood cells, causing secondary anemia, which can further aggravate the problem of microenvironment hypoxia. Our results were identical to those of Chen et al. [15], and the expression of HIF-1*α* mRNA in CML bone marrow patients was markedly elevated versus the control group.

2-Methoxyestradiol presents naturally in estrogen metabolites. It has been extensively studied as an anticancer agent. It has been indicated effective in inhibiting growth and inducing apoptosis of tumor cells including Ewing's sarcoma, chondrosarcoma, osteosarcoma, esophageal, hepatocellular, ovarian, nasopharyngeal, and prostate cancers in vivo and in vitro. To explore the role of HIF-1*α* in CML, 2-ME2 was employed as an experimental drug to decrease the HIF-1*α* expression. The CCK-8 and flow cytometry assays revealed that the change of HIF-1*α* expression affected the proliferation of K-562 cells, and this change was dose-dependent. As the concentration of 2-ME2 increased, the cell inhibition rate and cell apoptosis rate gradually increased. When the concentration was at least greater than 3.5 *μ*M, cell apoptosis increased. We speculated that 2-ME2 had accumulated to a certain concentration in K-562 cells at this time, which allowed it to play the roles of antiproliferation and apoptosis induction. The HIF-1*α* inhibitor 2-ME2 was applied at the concentration of IC50 of 9.4 *μ*mol/L against K-562 cells in vitro for 24 h. Its antiproliferative activity involves inhibiting HIF-1*α*, downregulating the antiapoptotic protein expressions of Bcl-xl and Bcl-2, thereby mediating cell apoptosis. To compare the therapeutic effects and combined effects of 2-ME2 and the previous targeted drug dasatinib, we investigated the antileukemia properties of 2-ME2 and dasatinib on K-562 cells. The experimental results showed that under the same condition, the sensitivity of K-562 cells to 2-ME2 was higher than that of dasatinib, and the therapeutic effect of 2-ME2 was significantly better than that of dasatinib. When both drugs were combined in the treatment, they could significantly increase the antiproliferation and apoptosis-inducing effects on leukemia cells. The findings indicated that 2-ME2 was a promising anticancer drug because it contributed effectively to inducing K-562 cell apoptosis. Unfortunately, the molecular mechanism of 2-ME2 inducing apoptosis has not been fully investigated. Given this doubt, protein expressions were sorted out from BCR-ABL downstream pathways. The results showed that 2-ME2 could markedly reduce the protein expression of C-myc.

C-myc gene serves as a protooncogene, which is a “switch” for cells to enter S phase from G0/G1 phase and a gene that promotes cell division [16]. The encoded protein links to various processes of cell proliferation, apoptosis, and differentiation. Abnormal C-myc expression exerts an essential function during processes of tumor occurrence and evolvement [17]. Myc represents a major transcription factor regulating multiple target genes. It is found to be altered in several types of cancers including solid tumors like bowel cancer, prostate cancer, breast cancer, and blood tumors like lymphoma. Tumors with highly expressed C-myc frequently manifest with a high proliferation rate, high invasiveness, high drug resistance, and poor prognosis [18]. C-myc gene expression was abnormally increased in different stages of CML: The levels in the blast phase were significantly higher than the accelerated phase, and the blast phase and the accelerated phase were markedly higher than the chronic phase and the control group. This result suggested that the high expression of C-myc gene may be one of the mechanisms of the progression and blast crisis of CML [19]. Additionally, tetrandrine can effectively induce leukemia cell differentiation and autophagy, and both ROS generation and C-myc inhibition are of pivotal significance in the process of autophagy and differentiation induced by tetrandrine [20]. It can be seen that C-myc can affect the occurrence and development of leukemia. Through the present experiment, when the concentration of 2-ME2 was at least greater than 0.5 *μ*M, the expression of C-myc in K-562 cells could be substantially inhibited, whereas the change in 2-ME2 concentrations did not affect the intensity of its downregulation of C-myc. A preliminary understanding of the findings indicated that 2-ME2 promoted the downregulation of C-myc by inhibiting the expression of HIF-1*α*, thereby accelerating cell division and inducing cell apoptosis.

The previously stated experimental results demonstrated that the abnormally high HIF-1*α* expression in K-562 cells is linked to the hypoxic environment in patients with CML. Inhibition of its expression helped to inhibit the proliferation and induce apoptosis of K-562 cells. Furthermore, when combined with dasatinib, it could induce apoptosis to a greater extent, and better therapeutic effects were achieved, which offers a new treatment insight for patients resistant to CML. However, in vivo experiments on the mechanism and therapeutic effects of 2-ME2 need to be further explored.

## 5. Conclusions

The bone marrow microenvironment of CML patients is under a highly hypoxic state, which is responsible for activating HIF-1*α* expression. The highly expressed HIF-1*α* exerted an essential role in CML occurrence and development. Inhibition of this gene could markedly increase cell apoptosis in a dose-dependent fashion. Furthermore, the therapeutic effect of 2-ME2 combined with dasatinib was superior to either of the single drug therapy. 2-ME2 could induce cell apoptosis by downregulating C-myc gene, and it exerted an apoptotic effect by downregulating antiapoptotic proteins Bcl-xl and Bcl-2.

## Data Availability

The data used to support the findings of this study are included within the article.
